# Induced redox responsiveness and electroactivity for altering the properties of micelles without external stimuli†
†Electronic supplementary information (ESI) available. See DOI: 10.1039/c4sm00258j



**DOI:** 10.1039/c4sm00258j

**Published:** 2014-04-16

**Authors:** Lidija Glavas, Karin Odelius, Ann-Christine Albertsson

**Affiliations:** a Fiber and Polymer Technology , School of Chemical Science and Engineering , KTH , Royal Institute of Technology , SE-100 44 Stockholm , Sweden . Email: aila@polymer.kth.se

## Abstract

Control over micelle properties is vital in the field of drug delivery, and the ability to modify these properties in order to trigger dissociation is highly desirable. We prepared polymeric micelles with the ability to undergo dissociation over time without the need for external stimulation by incorporating an electroactive and redox responsive segment into amphiphilic copolymers. The incorporation of this segment also provides the ability to tailor the critical micelle concentration (CMC) and micelle size of the copolymers. Amphiphilic PEG–PLA copolymers were functionalized by coupling to an aniline pentamer in two different oxidation states (leucoemeraldine and emeraldine state). The incorporation of the electroactive and redox responsive aniline pentamer decreased the CMCs and the micelle size, independent of the oxidation state. However, the copolymers with the aniline pentamer in the leucoemeraldine state had significantly lower CMCs than the copolymers with the aniline pentamer in the emeraldine state. Simultaneously, stability tests performed on the functionalized micelles demonstrated the oxidation of the aniline segment, from the leucoemeraldine to the emeraldine state, over time. The oxidation led to an increase in the CMC, and the copolymers could thereby represent an excellent starting point for triggering drug release without external stimuli.

## Abstract 2

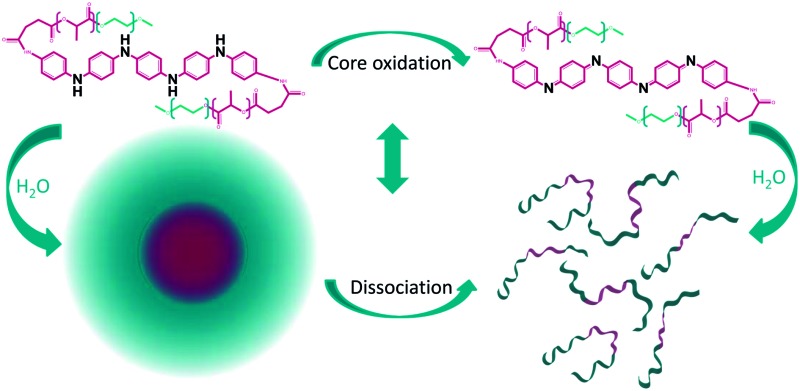
Triggered dissociation of micelles without external stimuli through incorporation of an aniline pentamer into amphiphilic PEG–PLA.

## Introduction

Micelles are a class of supramolecular structures that have been targeted for use as drug delivery systems.^1–3^ The aggregated structures are formed from amphiphilic copolymers due to a phase separation between the two distinctly different building blocks. The hydrophobic interactions act as the driving force for the self-assembly of micelles in water and induce the amphiphilic copolymers to form core–corona structures, where the core provides a lipophilic environment and is stabilized by the hydrophilic corona.^4,5^

Depending on the application, different building blocks can be used to prepare the self-assembling amphiphiles. PEG is commonly used as the hydrophilic block due to its hydrophilicity, biocompatibility, and stealth properties. The PEG block will help the micelles avoid protein adhesion while in the body, thereby prolonging their circulation time. The choice for the hydrophobic building block is more varied. Polylactide^6^ and poly(ε-caprolactone)^7^ have been used for a long time due to their intrinsic biocompatibility and biodegradability. However, both PLA and PCL lack functionality, and the ability to control the properties of PLA and PCL micelles is limited. To further control the micelle properties, additional building blocks can be added.^8,9^ The introduction of conjugated blocks can give rise to several desired properties, such as increased stability due to secondary bonding (π–π interactions).^10,11^ One example of these building blocks are the electro-responsive oligomers of aniline, which have been used for biomedical applications due to their ability to interact with cells.^12,13^ The oligomers possess electroactive properties similar to their respective polymer, but they also have a well-defined structure and are easy to prepare.^12,14,15^ The properties of the oligomers have further been altered by combining them with other polymers, such as PLA^16,17^ and PCL,^18^ to increase biocompatibility and biodegradability. This combination also facilitates the processing of the materials by enhancing their solubilities compared to the pure oligomers.

Well-defined micelle properties and good control over them are vital if the micelles are to be used as drug delivery systems. The size of the micelles determines their circulation time and their distribution throughout the body,^19–21^ while the critical micelle concentration (CMC) indicates the ability of the amphiphiles to form micelles and the stability of the micelles once they are formed. Therefore, it is crucial to gain control over these micelle properties.^4,22^ Several factors influence the CMC, such as the molecular weight of both of the building blocks^23^ and the crystallinity of the core.^24^ A low CMC is desirable in drug delivery applications to prevent the premature release of the drug due to the dissociation of the micelles; however, triggered dissociation of the micelles can be used as a mechanism for the drug release.^4,19^

Drug release is a key factor to consider for drug delivery systems and can occur through different mechanisms, including the degradation of the micelle core, leading to the disruption of the lipophilic environment,^25^ or diffusion through the core.^26^ Responsive micelles have proven to be an interesting route for controlled drug release due to their ability to respond to external stimuli. Drug release is then triggered by a change in the surrounding environment, such as a change in the pH^27^ or temperature,^28^ which often leads to the dissociation of the micelles, thereby releasing the drugs.^29^ Redox responsive micelles have been used for this exact purpose. For example, by introducing disulfide cross-linking, dissociation of the micelles can be triggered by the reductive environment in the cells.^30^ Near-infrared irradiation is another method used to trigger the dissociation of micelles to facilitate drug release.^31^

Triggered dissociation through stimuli is an effective way to release drugs and is highly desirable. However, if there is no change in the surrounding environment, such as a change in pH, when drug release is desired or if the drug delivery systems are out of the reach of external stimuli, triggered dissociation through external stimuli is ineffective. In these cases, systems that can undergo dissociation with time would be preferable.

The objective of this work was to expand the interchange between responsiveness and control over micelle properties by functionalizing amphiphilic biodegradable copolymers with electroactive segments. The introduction of an aniline pentamer will induce redox properties, as well as electroactivity, into the final material, which presents opportunities to alter the critical micelle concentration as a function of time, thereby enabling triggered drug release without external stimuli.

## Experimental

### Materials

l-Lactide (LA, Serva Feinbiochemica) was recrystallized from toluene twice, polyethylene glycol monomethyl ether (mPEG_2k_, Sigma-Aldrich) was dried under reduced pressure, and stannous 2-ethylhexanoate (Sn(Oct)_2_, ∼95%, Sigma-Aldrich) was dried over molecular sieves. Dicyclohexylcarbodiimide (DCC), 4-(dimethylamino)pyridine (DMAP), ammonium hydroxide, hydrochloric acid (37%), succinic anhydride, *p*-phenylenediamine, *N*-phenyl-*p*-phenylenediamine, and phenyl hydrazine (97%) were all obtained from Sigma-Aldrich and used without further purification; ammonium persulfate, ethanol, and dichloromethane were obtained from VWR. Dimethylformamide (DMF, HPLC, VWR) was dried over CaH_2_ and distilled under reduced pressure before use. Methanol (MeOH, HPLC, Fisher Scientific), trichloroacetyl isocyanate (96%, Sigma-Aldrich), 1,6-diphenyl-1,3,5-hexatriene (DPH, 98%, Sigma-Aldrich), chloroform (99%, Fisher Scientific), diethyl ether (99.8%, Sigma-Aldrich), anhydrous tetrahydrofuran (THF, Sigma-Aldrich), and hexamethylene diisocyanate (HMDI, 99%, Sigma-Aldrich) were all used without further purification.

### Polymer synthesis

Polymerization of PEG–PLA was performed in bulk at 110 °C for 48 h with 1 mol% of Sn(Oct)_2_ as the catalyst and mPEG_2k_ as the initiator. The copolymers were purified by precipitation in cold diethyl ether and were dried under reduced pressure. The carboxyl-capped aniline pentamer was prepared according to the literature.^32^ Briefly, the carboxyl-capped aniline dimer was oxidized by ammonium persulfate in the presence of phenylenediamine, yielding a carboxyl-capped aniline pentamer in the emeraldine state (EMAP). The aniline pentamer in the leucoemeraldine state (LMAP) was obtained by reducing EMAP with phenyl hydrazine. Mass spectrometry of the carboxyl-capped aniline pentamer yielded a peak at 673.5 (MH^+^/*z*). The coupling of the PEG–PLA copolymers with either LMAP or EMAP was performed by a condensation reaction in DMF at room temperature for 48 h using DCC as the condensing agent and DMAP as the catalyst. The unreacted aniline pentamer was removed by dissolution in chloroform, filtration, and then precipitation in cold diethyl ether. For comparison, a triblock copolymer (PEG_2k_–PLA–PEG_2k_) with a total *M*_n_ of 6000 was prepared by coupling the diblock copolymer (PEG–PLA) using HMDI as the coupling reagent. The coupling reaction was performed at 35 °C for 12 h in THF in an inert atmosphere and with a 2 : 1 ratio of PEG–PLA : HMDI.

### NMR spectroscopy

The chemical structures of the compounds were characterized by ^1^H-NMR in d-DMSO or CDCl_3_. The micelle formation was also confirmed by ^1^H-NMR using D_2_O as the solvent. The residual solvent peaks were used as internal standards: DMSO (*δ* = 2.50), CDCl_3_ (*δ* = 7.26), and H_2_O (*δ* ∼4.79). The ^1^H-NMR spectra were recorded on a Bruker Avance-400 nuclear magnetic resonance spectrometer at 400 MHz. The solutions in D_2_O were filtered using 0.45 μm nylon syringe filters before use in the NMR experiments.

### Size exclusion chromatography

Size exclusion chromatography (SEC) was used to obtain the number average molecular weight (*M*_n_) and dispersity (*Đ*) of each polymer. The measurements were performed on a TOSOH EcoSEC HLC-8320 GPC system equipped with an EcoSEC RI detector and three columns (PSS PFG 5 μm; Microguard, 100 Å and 300 Å) (*M*_W_ resolving range: 300–100 000 Da) from PSS GmbH, with 0.01 M LiBr in DMF (0.2 mL min^–1^) as the mobile phase at 50 °C. A conventional calibration method was performed using broad and narrow linear poly(methyl methacrylate) standards. Corrections for flow rate fluctuations were made using toluene as an internal standard.

### Laser desorption ionization mass spectroscopy

Laser desorption ionization mass spectroscopy (LDI-MS) was used to characterize the aniline pentamer. A 10 mg mL^–1^ solution of LMAP or EMAP in 1 M NH_4_OH was prepared and spotted onto the target plate. The analysis was performed on a Bruker UltraFlex time-of-flight mass spectrometer with a SCOUT-MTP ion source and a 337 nm nitrogen laser.

### Differential scanning calorimetry

The thermal properties of the copolymers were analyzed by differential scanning calorimetry (DSC) using a Mettler Toledo DSC 820 module. Four to eight milligrams of sample was placed in an aluminum cap (40 μL) and sealed with a lid. The atmosphere was kept inert during the experiment by a nitrogen flow of 50 mL min^–1^. The samples were first heated to 200 °C, kept isothermal for 10 min, cooled to –50 °C, kept isothermal for 10 min and finally heated to 200 °C. The rate of heating and cooling was 10 °C min^–1^. Samples of all of the copolymers were evaluated in triplicate, and an average value is presented with a moderate standard deviation. The melting enthalpies and temperatures were taken from the last heating cycle.

### Electroactivity measurements

The UV-vis spectra of the coupled copolymers in DMSO and H_2_O were recorded on a UV-vis spectrophotometer (UV-2401) directly after dissolution and then every week for 4 weeks. Cyclic voltammetry (CV) measurements of the copolymers coupled to the aniline pentamer in the leucoemeraldine state were performed on a potentiostat with a scan rate of 60 mV s^–1^. Platinum wires were used as the working and auxiliary electrodes, and a K/KCl reference electrode was used. The samples were prepared by dissolving 15 mg of the coupled copolymers in 6 mL of DMSO and doping the sample with 6 drops of 1 M HCl.

### Critical micelle concentration

The critical micelle concentration (CMC) was measured in micelle solutions prepared by direct dissolution at different concentrations from 0.0005–25 mg mL^–1^. To all of the micelle solutions, 10 μL mL^–1^ of a 0.4 mM DPH solution in MeOH was added.^33^ A UV-vis spectrum was then recorded on a UV-2401 UV-vis spectrophotometer.

### Dynamic light scattering

Micelle solutions were prepared using a dissolution/evaporation method.^34^ For example, 50 mg of the copolymer was dissolved in 5 mL of acetone, and 0.5 mL of the polymer solution was added dropwise to 10 mL of deionized water while stirring. Acetone was left to evaporate overnight, and dynamic light scattering (DLS) measurements were performed on a Malvern Zetasizer Nano ZS at 25 °C. All of the samples were filtered before measurement using 0.45 μm nylon syringe filters.

### Scanning transmission electron microscopy

The morphologies of the micelles were determined by scanning transmission electron microscopy using a FE-SEM (Hitachi S-4800, Japan) microscope equipped with a transmitted electron detector. The STEM sample was prepared by depositing a drop of a micelle solution onto a copper grid, and the water was left to evaporate slowly. Micelle solutions at concentrations of 0.5 mg mL^–1^ were prepared using a dissolution/evaporation method.

## Results and discussion

Functional micelles have been used as drug delivery vehicles due to their responsive nature and their ability for triggered drug release.^27,28^ However, the triggered release always occurs as an effect of external stimuli, such as a change in the pH or temperature. Therefore, it is highly desirable to prepare systems that are responsive without external stimuli. By attaching an aniline segment to the PEG–PLA copolymers, we can introduce electroactivity and redox properties into the final material, thereby obtaining the ability to alter micelle properties with time and without the influence of external stimuli.

### Polymer synthesis and characterization

**Fig. 1:**
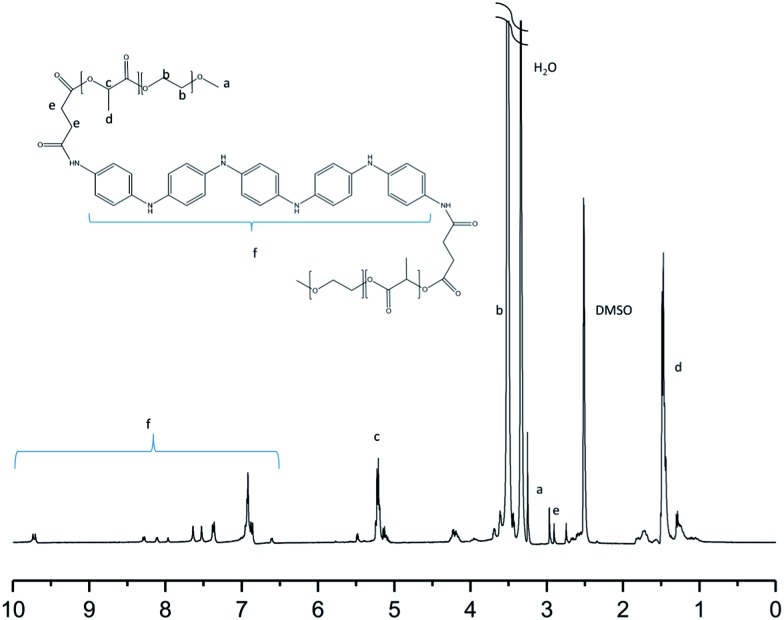
The ^1^H-NMR spectrum of (PEG_2k_–PLA_1k_)_2_–LMAP in DMSO.

Electroactivity was introduced into three amphiphilic PEG–PLA copolymers (*M*_n,tot_ = 3000, 4000 or 5000 g mol^–1^) by coupling them with an aniline pentamer in either the leucoemeraldine or emeraldine state through DCC/DMAP chemistry. The molecular weights obtained from the SEC and ^1^H-NMR analyses of the PEG–PLA copolymers corresponded well with the theoretical molecular weights (Table 1). Increases in the molecular weights of the coupled copolymers compared to the neat PEG–PLAs (Table 1), as well as the appearance of peaks corresponding to the aniline pentamer in the ^1^H-NMR spectrum at *δ* ∼ 7–10, indicated the successful incorporation of the aniline segment during coupling (Fig. 1).

**Table 1:** Characterization of the different copolymers in terms of their compositions and molecular weights, as determined by SEC and ^1^H-NMR, and their *Đ* values, as determined by SEC

Sample	*M*_n,Theo_ [g mol^–1^]	*M*_n_ ^*a*^ [g mol^–1^]	*M*_n_ ^*b*^ [g mol^–1^]	*Đ* ^2^
PEG_2k_–PLA_1k_	3000	2840	4030	1.1
(PEG_2k_–PLA_1k_)_2_–EMAP	6672	—^*c*^	5040	1.3
(PEG_2k_–PLA_1k_)_2_–LMAP	6672	—^*c*^	5630	1.2
PEG_2k_–PLA_2k_	4000	3450	5300	1.1
(PEG_2k_–PLA_2k_)_2_–EMAP	8672	—^*c*^	6490	1.3
(PEG_2k_–PLA_2k_)_2_–LMAP	8672	—^*c*^	7810	1.3
PEG_2k_–PLA_3k_	5000	4280	6080	1.2
(PEG_2k_–PLA_3k_)_2_–EMAP	10 672	—^*c*^	7060	1.3
(PEG_2k_–PLA_3k_)_2_–LMAP	10 672	—^*c*^	7790	1.3
PEG_2k_–PLA_2k_–PEG_2k_	5680	5510	4482	1.1

^*a*^The molecular weights were calculated from the ^1^H-NMR spectra by the integration of the methoxy peak of mPEG (at 3.37 ppm) and the peak for the repeating unit of LA (at 5.17 ppm).

^*b*^Determined by SEC using 0.01 M LiBr in DMF as an eluent.

^*c*^No calculation possible.

**Fig. 2:**
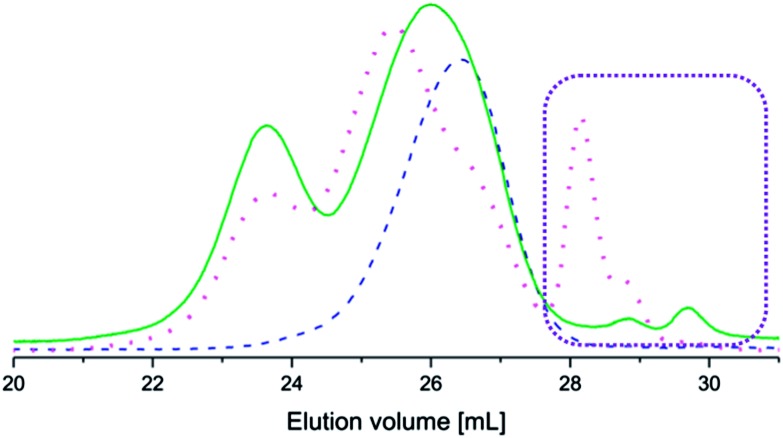
Size exclusion chromatograms of PEG_2k_–PLA_1k_ (dashed line), (PEG_2k_–PLA_1k_)_2k_–LMAP (dotted line) and (PEG_2k_–PLA_1k_)_2k_–EMAP (solid line). The consumption of EMAP was greater in the coupling reaction than the consumption of LMAP, as indicated by the rectangle.

The coupled copolymers exhibited bimodal populations as observed by SEC (Fig. 2) due to side reactions that occurred during coupling.^35^ The side reactions also resulted in unreacted aniline pentamer, which was removed from the coupled copolymers by dissolution in CHCl_3_ (a non-solvent for the aniline pentamer), followed by filtration. It has been shown that the bimodal nature of the coupled copolymers could lead to enhanced stability in the resulting micelles.^36^

The coupling reactions with EMAP reached higher conversions than the reactions with LMAP, as indicated by the difference in peak height of the residual aniline pentamer in the SEC chromatograms (Fig. 2). These results also indicated that the hydrodynamic volume of the aniline pentamer in the emeraldine state is smaller than that of the aniline pentamer in the leucoemeraldine state. One explanation for this phenomenon is that the aniline pentamer takes on a different conformation in the emeraldine state and, therefore, has a smaller hydrodynamic volume. This trend is observed for the coupled copolymers as well, as the copolymers with EMAP had lower molecular weights (obtained from SEC) than the copolymers with LMAP.

For comparison, a triblock copolymer (PEG_2k_-PLA_2k_–PEG_2k_) was prepared by the coupling of PEG_2k_–PLA_1k_ with HMDI. The SEC and NMR results confirmed the formation of the triblock copolymer (Table 1).

### Thermal properties

All of the polymers exhibited two crystalline phases corresponding to the crystallization of the PEG block and the PLA block (ESI†). An exception was the neat PEG_2k_–PLA_1k_, of which only the PEG block melted. This is most likely due to the PEG block interfering with the crystallization of the low molecular weight PLA. However, as the molecular weight of the PLA block increases, the interference of the PEG block decreases, allowing the PLA block to crystallize and simultaneously interfere with the crystallization of the PEG block, which lowers the melting enthalpy of PEG.

The incorporation of the aniline segment did not lead to a noticeable change in the melting enthalpy of the PLA block. Thus, the introduction of the aniline segment does not interfere with or induce the crystallization of PLA to a large extent. However, the melting enthalpy of the PEG block decreased with the incorporation of the aniline segment, indicating that it influences the crystallization of PEG. The crystallization of the copolymers is complex and is affected by many different factors, such as the composition and molecular weight of each block. The melting enthalpy for the blocks is, therefore, only used as an indication of the crystallinity of the blocks.

The obtained melting enthalpies should be considered as indicators of the thermal properties of the polymers and will change slightly when in micellar solutions; for example, the PEG block will dissolve and, therefore, not crystallize. Measurements of the PEG–PCL micelles in water have previously shown that the crystallization of the core does occur while the PEG block is completely dissolved.^37^

### Electrochemical behavior

**Fig. 3:**
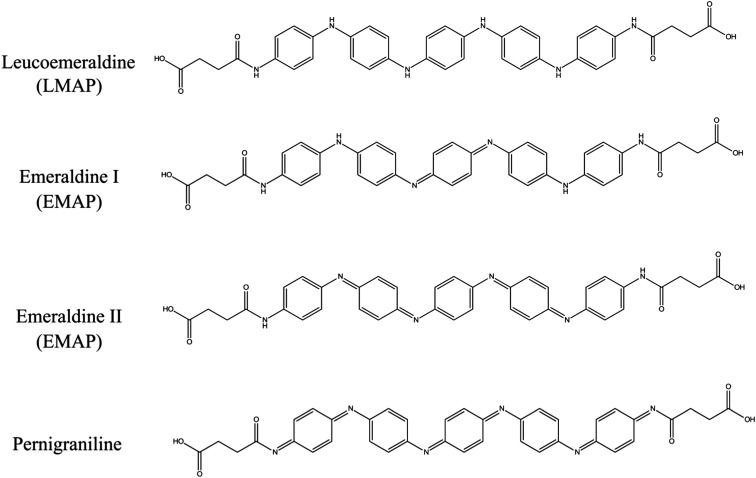
The chemical structures of the aniline pentamer in various oxidation states.

In order for a material to be electroactive, the electrons need to be able to move within the material. This is enabled by the oxidation or reduction of the material. The aniline pentamer has the ability to undergo both chemical and electrochemical oxidation or reduction due to its conjugated system, and the introduction of this segment into PEG–PLA will, therefore, induce electroactivity into the final copolymers. There are four different oxidation states of the aniline segment: the fully reduced leucoemeraldine (LMAP), the emeraldine I (EMAP I), the emeraldine II (EMAP II) and the fully oxidized pernigraniline state (Fig. 3).^32,38,39^ The final copolymers were prepared with the fully reduced LMAP or the partially oxidized EMAP.

**Fig. 4:**
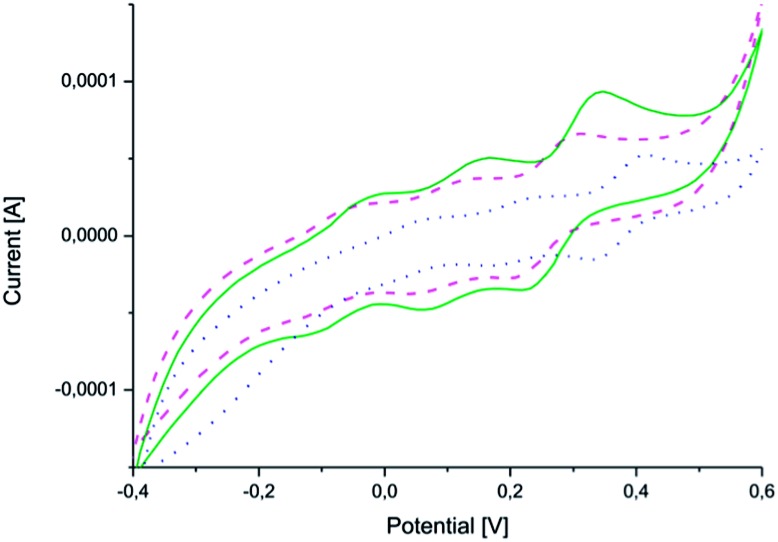
Cyclic voltammograms of (PEG_2k_–PLA_1k_)_2_–AP (solid line – green), (PEG_2k_–PLA_2k_)_2_–AP (dashed line – pink) and (PEG_2k_–PLA_3k_)_2_–AP (dotted line – blue).

CV measurements (Fig. 4) confirmed that the copolymers were electroactive and that they retained their ability to undergo oxidation and reduction after coupling (Fig. 4). The measurements also confirmed the reversibility of oxidation and reduction processes of the electroactive segment.

All three copolymers exhibit three characteristic redox peaks for the aniline pentamer. The first redox peak corresponds to the transition from the leucoemeraldine state to the emeraldine I state, the second from the emeraldine I state to the emeraldine II state and the last from the emeraldine II state to the fully oxidized pernigraniline state.^32,38^ The oxidation/reduction behavior of the aniline pentamer remained after coupling and can, therefore, be used to induce changes in the final properties of the polymers.

**Fig. 5:**
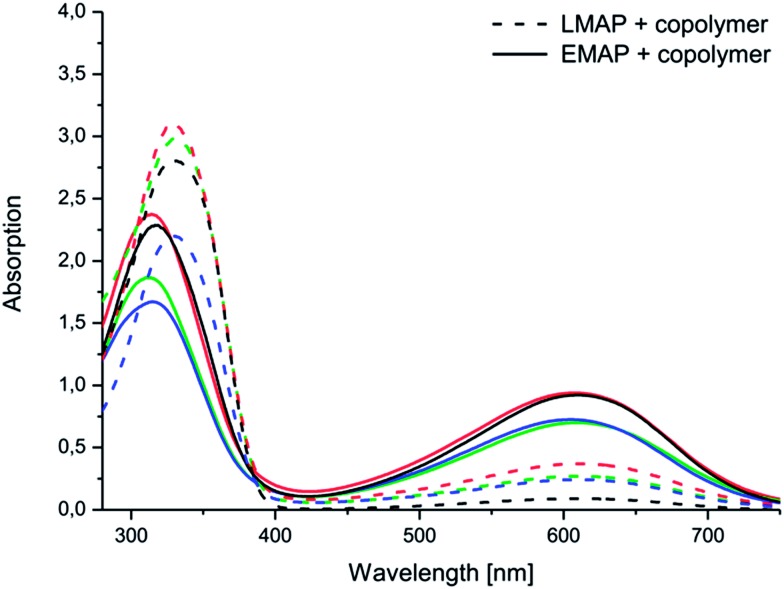
Electroactivity of the aniline pentamer (black) and the following coupled copolymers: (PEG_2k_–PLA_1k_)_2_–AP (green), (PEG_2k_–PLA_2k_)_2_–AP (red) and (PEG_2k_–PLA_3k_)_2_–AP (blue) in DMSO, as measured by UV-vis spectroscopy.

Differences between the copolymers with LMAP and those with EMAP were clearly observed in the UV spectra (Fig. 5), as the copolymers with LMAP only exhibited one distinct peak, and the copolymers with EMAP exhibited two distinct peaks. The π–π* transition of the benzene rings of LMAP gave rise to an absorption peak at 329 nm, while the same transition for EMAP was found at 316 nm with a lower intensity. For EMAP, another peak appeared at 610 nm, which was attributed to the transition of the benzene rings to quinoid rings.^12^

The aniline segment was prepared in the desired oxidation state and then coupled to PEG–PLA. During the coupling reaction, EMAP retained its oxidation state, while LMAP was slightly oxidized, which was indicated by the beginning of a peak at ∼600 nm. However, the difference between the copolymers with LMAP and EMAP was still clear and significant, and only a small amount of LMAP was oxidized.

**Fig. 6:**
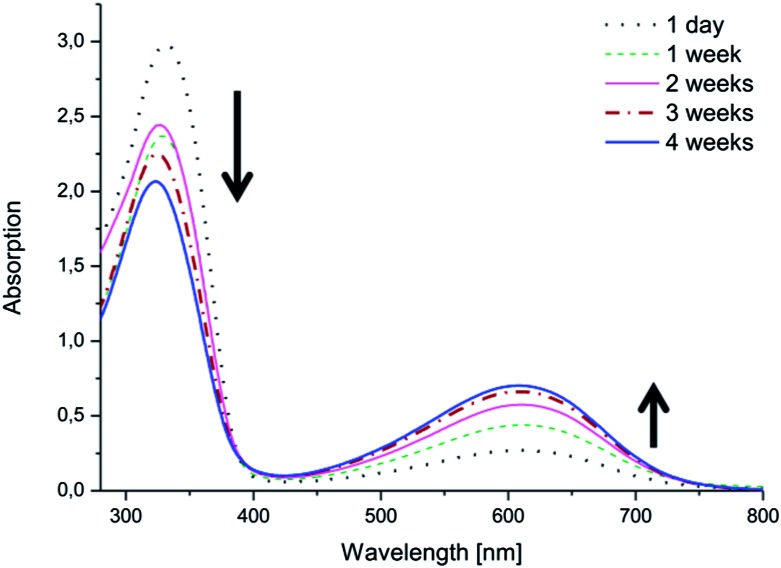
The electroactivity of (PEG_2k_–PLA_1k_)_2_–LMAP in DMSO over time, as measured by UV-vis spectroscopy.

Changes in the electroactivities of the copolymers were observed over time. While the copolymers with EMAP exhibited good stability and retained their electroactivity and oxidation state, the copolymers with LMAP displayed a different behavior (Fig. 6 and ESI†). The peak at ∼610 nm, which was initially barely visible for the copolymers with LMAP, started to increase in intensity with time, as the π–π* transition of the benzene rings (∼320 nm) simultaneously decreased in intensity and shifted slightly. This indicates that LMAP in the copolymers oxidized to EMAP over time in DMSO.

**Fig. 7:**
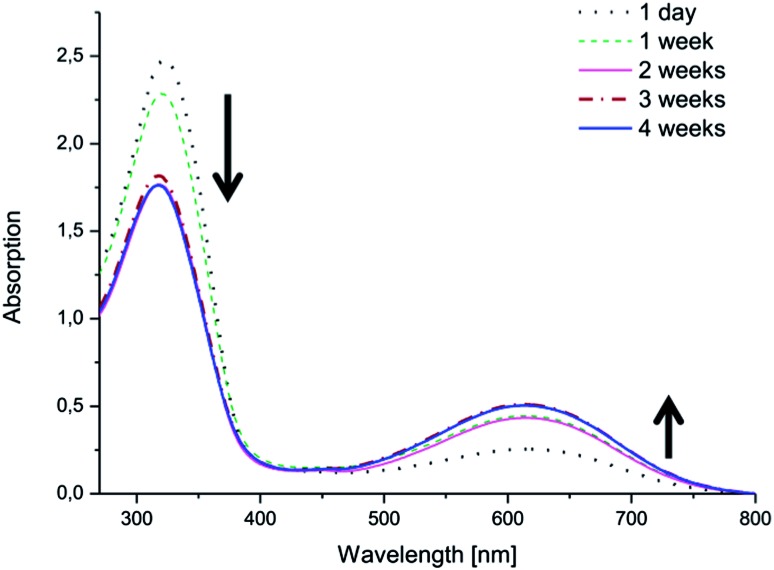
The electroactivity of (PEG_2k_–PLA_1k_)_2_–LMAP in H_2_O over time, as measured by UV-vis spectroscopy.

The same phenomenon was observed in water (Fig. 7 and ESI†). The copolymers with LMAP exhibited oxidation after 4 weeks in solution. The oxidation in water was dependent on the molecular weight of the lactide block and occurred more readily for the copolymers with lower molecular weights. Thus, (PEG_2k_–PLA_1k_)_2_–LMAP was most readily oxidized, while (PEG_2k_–PLA_3k_)_2_–LMAP exhibited the least oxidation.

The concentration of the copolymer in the water samples was higher than the copolymer concentration in the DMSO samples; however, the absorption intensity of the copolymer was lower in the water samples. The oxidation also exhibited a molecular weight dependence which was not observed in DMSO.

### Micelle characterization

**Fig. 8:**
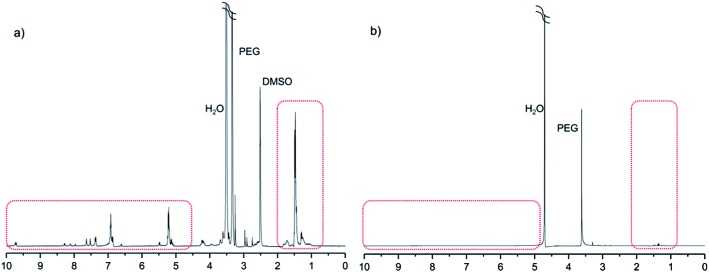
Evidence of the self-assembly of (PEG_2k_–PLA_1k_)_2_–LMAP. ^1^H-NMR was performed in (a) DMSO and (b) D_2_O.

All of the prepared copolymers had the ability to self-assemble into micelles, which was demonstrated by ^1^H-NMR in D_2_O. The spectra of the copolymers in D_2_O only exhibited peaks for the PEG block, indicating that self-assembly occurred, with PEG acting as a protecting corona and shielding the hydrophobic core, which consisted of PLA and AP (Fig. 8). The micelle formation was further confirmed by STEM (ESI†).

### Critical micelle concentration

The introduction of a new segment into the copolymer either in the hydrophobic or the hydrophilic block will subsequently influence the micelle properties. For example, by coupling a hydrophilic molecule, such as cyclodextrin, to an amphiphilic copolymer, an increase in the CMC will be obtained.^40^ Here, when we introduce an aniline segment into the core of a micelle to induce electroactivity into the final copolymer, we simultaneously influence the micelle properties.

**Fig. 9:**
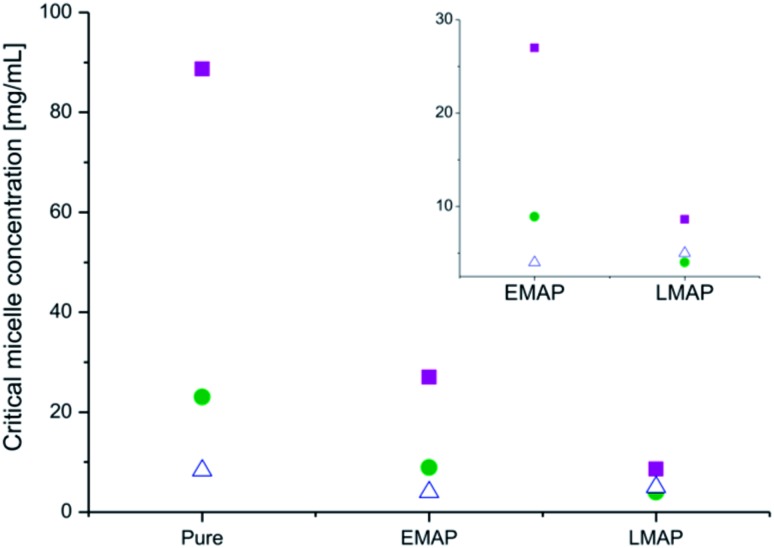
The change in the CMCs after the addition of the aniline pentamer and the change in its oxidation state ((PEG_2k_–PLA_1k_)_2_–AP (■), (PEG_2k_–PLA_2k_)_2_–AP () and (PEG_2k_–PLA_3k_)_2_–AP (△)) in DMSO.

A decrease in the CMC, with an increase in the molecular weight of the hydrophobic block, was observed for all of the copolymers, as expected (Fig. 9). This decrease is due to an increase in surface tension that accompanies a larger hydrophobic block.^41,42^

The introduction of the aniline pentamer into the copolymer gave rise to a lower CMC, regardless of the oxidation state of the segment, compared to the diblock and the triblock copolymers without the aniline segment (ESI†). Generally, a higher CMC is observed for triblock copolymers with flanking hydrophilic blocks than for their diblock precursors.^43^ The aniline pentamer contributes to the hydrophobic block, and thus, the coupling will increase the molecular weight of the hydrophobic block, increase the hydrophobic ratio and decrease the CMC. The induced π–π interactions that occur due to the conjugated system of the aniline segments also contribute to the decrease in the CMC.

The extent to which the CMC is decreased is dependent on the oxidation state of the aniline segment. When the aniline segment in the copolymer is in its fully reduced form (LMAP), the micelles have a lower CMC than when the aniline segment is partly oxidized (EMAP). This is most likely due to the difference in the number of hydrogen bonding sites in the different oxidation states. LMAP has six amines that can act as hydrogen bonding donors, while EMAP has only two. This decrease reduces the amount of secondary bonds stabilizing the core, lowers the stability of the micelles and increases the CMC values. The effect of hydrogen bonding (differences in the CMCs between LMAP and EMAP) decreases with an increase in the molecular weight of the hydrophobic block. This is due to the effect of surface tension becoming much more prevalent than the effect of hydrogen bonding, with an increase in the molecular weight of the hydrophobic block.

**Fig. 10:**
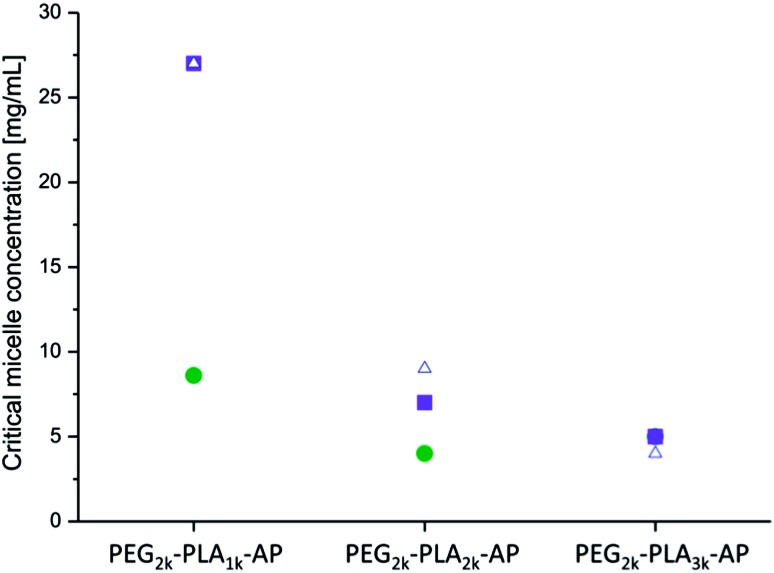
The CMC of PEG–PLA–LMAP micelles after 1 day (), PEG–PLA–LMAP micelles after 1 week (■) and PEG–PLA–EMAP micelles after 1 day (△).

UV measurements indicated that if the copolymers were left to stand in solution, the aniline segment in the copolymers oxidized from LMAP to EMAP. Copolymers with LMAP give rise to micelles with low CMCs; however, over time the aniline segment will oxidize, and the CMC will increase. This was confirmed by measuring the CMCs of the copolymers with LMAP after one week in water. The results demonstrate a considerable increase in the CMC over time (Fig. 10), which corresponds to the oxidation of the aniline segment. This increase is the largest for the polymers with the lowest total molecular weight (∼6000 g mol^–1^), and this effect decreases with increasing molecular weights. This is explained by a decrease in the amount of the aniline segment with an increase in the molecular weight of the copolymer, and therefore, the influence of the oxidation decreases as well. If formulated correctly, this increase in the CMC could be used to trigger the dissociation of the micelles and release drugs after a specified time. Optimization of the hydrophobic ratio to induce a larger difference in the CMC between the copolymers with LMAP and the copolymers with EMAP could lead to controlled disassembly with time as the aniline segment oxidizes. The molecular weight did not notably decrease during a month in water and, therefore, should not influence the changes observed in the CMC over time (ESI†).

### Micelle size

The micelle size is an important property because it determines the circulation time as well as the distribution of the micelles in the body. Smaller micelles have the ability to accumulate in tumors due to the enhanced permeability and retention (EPR) effect.

Most of the polymers showed a bimodal distribution in DLS measurements, indicating the existence of two populations with different sizes (ESI†). This can be explained by the aggregation phenomenon; the micelles are not stable by themselves and, therefore, aggregate into larger particles, giving rise to a population with a bigger size. This has been previously observed for linear PEG–PLA copolymers.^44^

**Fig. 11:**
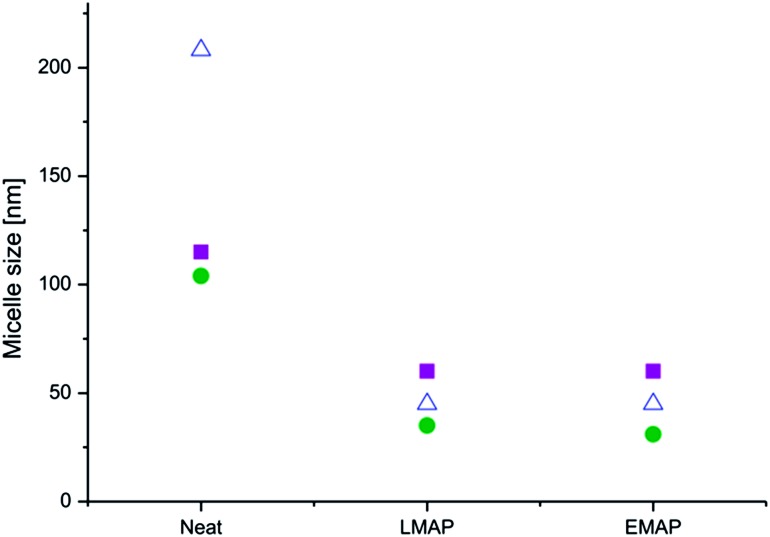
The micelle size as a function of the core composition at a concentration of 0.5 mg mL^–1^ ((PEG_2k_–PLA_1k_)_2_–AP (■), (PEG_2k_–PLA_2k_)_2_–AP () and (PEG_2k_–PLA_3k_)_2_–AP (△)).

The incorporation of the aniline pentamer, either in the leucoemeraldine or the emeraldine state, decreased the micelle size (Fig. 11). This could be due to the addition of secondary interactions introduced by the aniline pentamer in the core, which makes the core more compact and reduces its size. The aniline pentamer gives rise to hydrogen bonding and π–π interactions that could work to reduce the distance between the chains, making the core smaller. The biggest decrease in the micelle size with the incorporation of the aniline pentamer was observed with the highest molecular weight copolymers, and the smallest difference was observed for the lowest molecular weight copolymers. There was no distinct difference in size between the micelles in the leucoemeraldine and emeraldine state.

## Conclusions

Successful tailoring of the critical micelle concentration (CMC) and the micelle size was achieved by preparing electroactive and redox responsive micelles containing aniline segments. The addition of an aniline segment to PEG–PLA copolymers caused a reduction in the CMC by up to a factor of 10. The largest reduction was obtained using the fully reduced form of the aniline pentamer. The copolymers with the fully reduced aniline pentamer underwent oxidation in solution over time without external stimuli. The CMC increased with the oxidation of the aniline pentamer, and the oxidation effect could, therefore, be further used to trigger the dissociation of the micelles.

A decrease in the micelle size was obtained by the incorporation of the aniline segment. The size was not dependent on the oxidation state of the aniline segment but instead depended on its hydrophobicity. The ability to change the properties of micelles over time without outside influence is one of the next important steps in optimizing drug delivery systems.

## References

[cit1] Kabanov A. V., Batrakova E. V., Melik-Nubarov N. S., Fedoseev N. A., Dorodnich T. Y., Alakhov V. Y., Chekhonin V. P., Nazarova I. R., Kabanov V. A. (1992). J. Controlled Release.

[cit2] Zhang S., Zou J., Elsabahy M., Karwa A., Li A., Moore D. A., Dorshow R. B., Wooley K. L. (2013). Chem. Sci..

[cit3] Kataoka K., Matsumoto T., Yokoyama M., Okano T., Sakurai Y., Fukushima S., Okamoto K., Kwon G. S. (2000). J. Controlled Release.

[cit4] Aseyev V., Tenhu H. and Winnik F. M., in Self Organized Nanostructures of Amphiphilic Block Copolymers II, ed. A. H. E. Muller and O. Borisov, Springer-Verlag Berlin, Berlin, 2011, vol. 242, pp. 29–89.

[cit5] Cameron N. S., Corbierre M. K., Eisenberg A. (1999). Can. J. Chem..

[cit6] Hagan S. A., Coombes A. G. A., Garnett M. C., Dunn S. E., Davies M. C., Illum L., Davis S. S., Harding S. E., Purkiss S., Gellert P. R. (1996). Langmuir.

[cit7] Allen C., Han J., Yu Y., Maysinger D., Eisenberg A. (2000). J. Controlled Release.

[cit8] Sun T.-M., Du J.-Z., Yan L.-F., Mao H.-Q., Wang J. (2008). Biomaterials.

[cit9] Chen W., Zou Y., Jia J., Meng F., Cheng R., Deng C., Feijen J., Zhong Z. (2013). Macromolecules.

[cit10] Cui H., Shao J., Wang Y., Zhang P., Chen X., Wei Y. (2013). Biomacromolecules.

[cit11] Xiong W., Wang H., Han Y. (2011). Soft Matter.

[cit12] Huang L., Hu J., Lang L., Wang X., Zhang P., Jing X., Wang X., Chen X., Lelkes P. I., MacDiarmid A. G. (2007). Biomaterials.

[cit13] Cao J., Man Y., Li L. (2013). Biomed. Rep..

[cit14] Guo B., Glavas L., Albertsson A.-C. (2013). Prog. Polym. Sci..

[cit15] Zhang W., Feng J., MacDiarmid A., Epstein A. (1997). Synth. Met..

[cit16] Huang L., Zhuang X., Hu J., Lang L., Zhang P., Wang Y., Chen X., Wei Y., Jing X. (2008). Biomacromolecules.

[cit17] Guo B., Finne-Wistrand A., Albertsson A.-C. (2011). Chem. Mater..

[cit18] Guo B., Finne-Wistrand A., Albertsson A.-C. (2011). Macromolecules.

[cit19] Liu J., Zeng F., Allen C. (2007). Eur. J. Pharm. Biopharm..

[cit20] Stolnik S., Illum L., Davis S. S. (1995). Adv. Drug Delivery Rev..

[cit21] Kwon G., Yokoyama M., Okano T., Sakurai Y., Kataoka K. (1993). Pharm. Res..

[cit22] Allen C., Maysinger D., Eisenberg A. (1999). Colloids Surf., B.

[cit23] Alexandridis P., Athanassiou V., Fukuda S., Hatton T. A. (1994). Langmuir.

[cit24] Glavas L., Olsén P., Odelius K., Albertsson A.-C. (2013). Biomacromolecules.

[cit25] Samarajeewa S., Shrestha R., Li Y., Wooley K. L. (2011). J. Am. Chem. Soc..

[cit26] Soo P. L., Luo L. B., Maysinger D., Eisenberg A. (2002). Langmuir.

[cit27] Jiang X., Zhao B. (2008). Macromolecules.

[cit28] Wan X., Liu T., Liu S. (2011). Biomacromolecules.

[cit29] Dong J., Wang Y., Zhang J., Zhan X., Zhu S., Yang H., Wang G. (2013). Soft Matter.

[cit30] Yue J., Wang R., Liu S., Wu S., Xie Z., Huang Y., Jing X. (2012). Soft Matter.

[cit31] Yan B., Boyer J.-C., Branda N. R., Zhao Y. (2011). J. Am. Chem. Soc..

[cit32] Huang L., Hu J., Lang L., Wang X., Zhang P., Jing X., Wang X., Chen X., Lelkes P. I., MacDiarmid A. G., Wei Y. (2007). Biomaterials.

[cit33] Kabanov A. V., Nazarova I. R., Astafieva I. V., Batrakova E. V., Alakhov V. Y., Yaroslavov A. A., Kabanov V. A. (1995). Macromolecules.

[cit34] Shuai X., Ai H., Nasongkla N., Kim S., Gao J. (2004). J. Controlled Release.

[cit35] Moore J. S., Stupp S. I. (1990). Macromolecules.

[cit36] Lo C.-L., Lin S.-J., Tsai H.-C., Chan W.-H., Tsai C.-H., Cheng C.-H. D., Hsiue G.-H. (2009). Biomaterials.

[cit37] Glover A. L., Nikles S. M., Nikles J. A., Brazel C. S., Nikles D. E. (2012). Langmuir.

[cit38] Chen L., Yu Y., Mao H., Lu X., Zhang W., Wei Y. (2005). Synth. Met..

[cit39] Pouget J. P., Laridjani M., Jozefowicz M. E., Epstein A. J., Scherr E. M., MacDiarmid A. G. (1992). Synth. Met..

[cit40] Chen H., Chen W., Zheng W., Lei Z., Li H. (2013). Int. J. Polym. Mater..

[cit41] Wilhelm M., Zhao C. L., Wang Y., Xu R., Winnik M. A., Mura J. L., Riess G., Croucher M. D. (1991). Macromolecules.

[cit42] Astafieva I., Khougaz K., Eisenberg A. (1995). Macromolecules.

[cit43] Booth C., Attwood D. (2000). Macromol. Rapid Commun..

[cit44] Yang L., Zhao Z., Wei J., El Ghzaoui A., Li S. (2007). J. Colloid Interface Sci..

