# Supplementary material for: Induced redox responsiveness and electroactivity for altering the properties of micelles without external stimuli
Source: Soft Matter. 2014 Apr 16;10(22):3859–60. doi: 10.1039/c4sm00258j (PMC4159694; doi:10.1039/c4sm00258j)
Supplement: Supplementary file 1 [file SM-010-C4SM00258J-s001.pdf]

Supporting information

Induced redox responsiveness and electroactivity for altering micelle properties without external stimuli

*Lidija Glavas, Karin Odelius, Ann-Christine Albertsson**

Fiber and Polymer Technology, School of Chemical Science and Engineering, KTH, Royal Institute of Technology, SE-100 44 Stockholm, Sweden

Thermal properties

Table 1. Thermal properties of the copolymers as determined by DSC.

Sample name	ΔH $_{PEG}$ [J/g]	$T_{m, PEG}$ [°C]	ΔH $_{PLA}$ [J/g]	$T_{m, PLA}$ [°C]
PEG_{2k}-PLA_{1k}	103	47	-	-
(PEG_{2k}-PLA_{1k})$_2$-EMAP	96	48	-	-
(PEG_{2k}-PLA_{1k})$_2$-LMAP	74	47	-	-
PEG_{2k}-PLA_{2k}	70	45	12	128
(PEG_{2k}-PLA_{2k})$_2$-EMAP	58	43	10	122
(PEG_{2k}-PLA_{2k})$_2$-LMAP	42	39	11	124
PEG_{2k}-PLA_{3k}	46	42	21	145
(PEG_{2k}-PLA_{3k})$_2$-EMAP	41	41	25	153
(PEG_{2k}-PLA_{3k})$_2$ -LMAP	37	37	22	142

Table 2. The molecular weights of EMAP copolymer directly after synthesis and LMAP copolymer after 1 week in water as determined by SEC.

Sample name	$M_{n, before}$ [g/mol]	$M_{n, after\ 1\ week}$ [g/mol]
(PEG_{2k}-PLA_{1k})$_2$-AP	5040	5100
(PEG_{2k}-PLA_{2k})$_2$-AP	6490	6780
(PEG_{2k}-PLA_{3k})$_2$ -AP	7060	7860

Electrochemical behavior

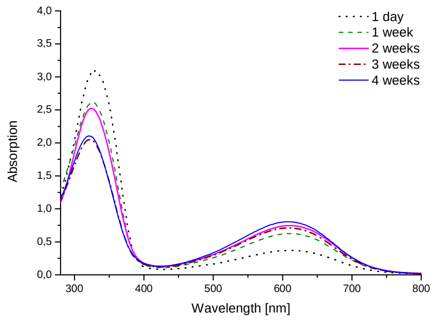

Figure 1. The electroactivity of the (PEG_{2k}-PLA_{2k})$_2$-LMAP in DMSO with time.

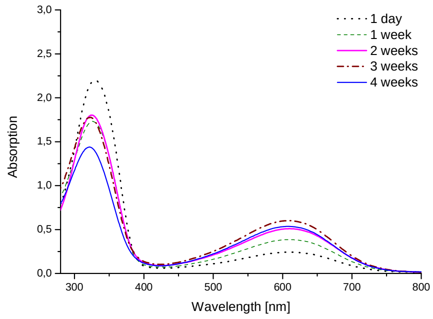

Figure 2. The electroactivity of the (PEG_{2k}-PLA_{3k})$_2$-LMAP in DMSO with time.

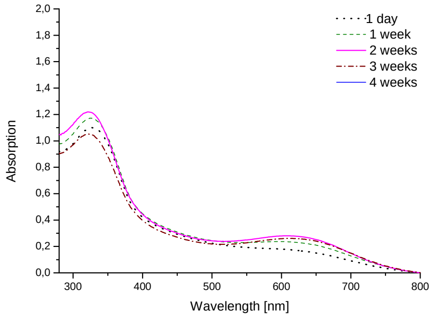

Figure 3. The electroactivity of the $(PEG_{2k}$-$PLA_{3k})_2$-LMAP in H_2O with time.

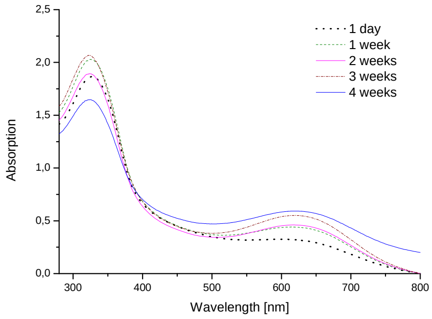

Critical micelle concentration

Table 3. The CMC values of the prepared copolymers.

Sample name	CMC [mg/L]
PEG_{2k}-PLA_{1k}	88
$(PEG_{2k}$-$PLA_{1k})_2$-EMAP	27
$(PEG_{2k}$-$PLA_{1k})_2$-LMAP	9
PEG_{2k}-PLA_{2k}	23
$(PEG_{2k}$-$PLA_{2k})_2$-EMAP	9
$(PEG_{2k}$-$PLA_{2k})_2$-LMAP	4
PEG_{2k}-PLA_{3k}	8
$(PEG_{2k}$-$PLA_{3k})_2$-EMAP	4
$(PEG_{2k}$-$PLA_{3k})_2$ -LMAP	5

PEG$_{2k}$-*PLA*$_{2k}$-*PEG*$_{2k}$	380

Micelle size

Table 4. The micelle size and PDI of the prepared copolymers.

Sample name	Size [nm]	PDI
PEG_{2k}-PLA_{1k}	115	0.2
PEG_{2k}-PLA_{1k}/LMAP	60	0.3
PEG_{2k}-PLA_{1k}/EMAP	60	0.4
PEG_{2k}-PLA_{2k}	104	0.6
PEG_{2k}-PLA_{2k}/LMAP	35	0.2
PEG_{2k}-PLA_{2k}/EMAP	31	0.4
PEG_{2k}-PLA_{3k}	208	0.3
PEG_{2k}-PLA_{3k}/LAMP	45	0.3
PEG_{2k}-PLA_{3k}/EMAP	45	0.2

Morphology

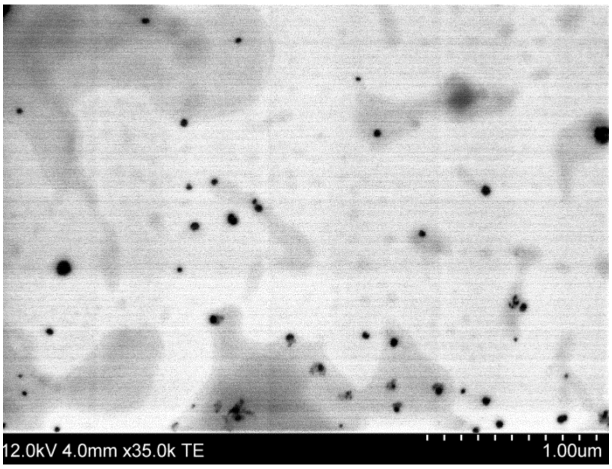

Figure 4. STEM micrograph of $(PEG_{2k}$-$PLA_{1k})_2$-EMAP.
